# Social return on investment (SROI) method to evaluate physical activity and sport interventions: a systematic review

**DOI:** 10.1186/s12966-020-00931-w

**Published:** 2020-02-27

**Authors:** Véronique Gosselin, Dorothée Boccanfuso, Suzanne Laberge

**Affiliations:** 1 École de kinésiologie et des sciences de l’activité physique, Faculté de médecine, Université de Montréal, C.P. 6128, succursale Centre-ville,, Montréal, Québec H3C 3J7 Canada; 2 Faculté de Gouvernance, Sciences Économiques et Sociales, Université Mohammed VI Polytechnique, Lot 660 - Hay Moulay Rachid, 43150 Ben Guerir, Morocco

**Keywords:** Physical activity, Sports, Social return on investment, Economic evaluation, Social impact, Systematic review

## Abstract

**Background:**

Physical Activity and Sport (PAS) interventions can reduce the social and economic burden of non-communicable diseases and improve the wellbeing of the population. Social return on investment (SROI) has the capacity to measure broader socio-economic outcomes in a singular monetary ratio to help identify the most impactful and cost-beneficial intervention. This review aimed to systematically identify and review studies using the SROI method within the field of PAS and assess their quality.

**Methods:**

Peer-reviewed and grey literature SROI studies were identified through a systematic search of six databases. Two reviewers independently assessed the identified studies to determine eligibility. Study quality was assessed using the Krelv et al. 12-point framework. For each included study, information was extracted and classified into summary tables. Extracted information included study and participant characteristics, type of outcomes and SROI ratio. The PRISMA guidelines were followed.

**Results:**

Seventeen studies published between 2010 and 2018 met the inclusion criteria. Most studies (94%) were non-peer reviewed publicly available reports, primarily conducted in the UK (76%), by private consulting firms (41%) and included all types of stakeholders (76%). PAS interventions included Primary prevention (47%), Sport for development (29%), Secondary and tertiary prevention (18%) and High-performance sport (6%). SROI ratios, which report the social value created in relation to the cost of an intervention, vary between 3:1 and 124:1 for the high-quality studies.

**Conclusions:**

The SROI framework can be a useful tool to inform policy-making relating to PAS investment as it can account for the wide societal benefits of PAS. The quality of studies in the field would benefit from the employment of an impact map (or logic model), reporting negative outcomes and using objective study designs. The application of the SROI method in the PAS field is relatively recent, and thus further research would be beneficial to promote its potential for policy-making bodies in the field.

## Background

Regular physical activity is proven to help prevent and treat noncommunicable diseases (NCDs) such as heart disease, stroke, hypertension, diabetes and breast and colon cancer. It can also help to prevent weight-gain and obesity, while also contributing to improved mental health, quality of life and well-being [1, 2]. In addition to these health benefits, there is a growing body of literature measuring the ways in which physical activity and sport (PAS) contribute to society [3]. For example, Taylor et al. [4], in a systematic review on the social impact of culture and sport, provide evidence of the impact of sport on crime reduction, educational achievement, subjective well-being, and social capital. Economic productivity and civic participation have also been identified as social contributions of PAS [1, 4, 5]. As a strategy to reduce the social and economic burden of NCDs, improve the wellbeing of the population and benefit from their broad contribution to society, many countries, through different levels of governance, are investing in different PAS interventions [6–9]. For instance, in the United Kingdom (UK), the five outcomes identified in *Sporting Future, the Government’s strategy for sport* are physical wellbeing, mental wellbeing, individual development, social and community development and economic development [10]. In Quebec, 64 million CAD was recently invested in different interventions intended to promote PAS, which include a school-based PAS policy. The policy target is to provide 60 min of daily PAS to all children, with several expected benefits including improved cognitive skills, educational achievement, wellbeing, physical and mental health, as well as more developed social and relational skills [11]. Economic evaluations can be used to determine whether policies and interventions achieve the highest attainable impacts and to inform the policymaking process. Traditional frameworks of economic evaluations include cost-effectiveness analysis (CEA), cost-utility analysis (CUA) and cost-benefit analysis (CBA). Over the past decade, the social return on investment (SROI) framework has received increased attention within the public health domain [12–16].

SROI is a framework used for understanding, measuring, and reporting the social, economic and environmental value created by an intervention, programme, policy or organisation [17]. It has its foundations in traditional economic evaluation [16, 18, 19] and is recognized for providing a holistic framework in its inclusion of entire social impact and a strong engagement with the stakeholders [13, 15, 17, 18, 20].

The SROI provides an indication of the efficiency of an investment by comparing the value of its benefits to the value of the resources invested in order to assess comparative options*.* To do so, it uses monetary values to represent the social value created by an intervention by accounting for the whole range of the value generated, beyond a narrow microeconomic dimension [21, 22]. This allows for the calculation of a benefits to costs ratio [17]. For example, a ratio of 2:1 indicates that an investment of $1 delivers $2 of social value.

There are two types of SROI: evaluative (conducted after an intervention and based on outcomes that have already taken place) and forecast (conducted before an intervention to predict how much social value will be created if it meets the intended outcomes) [17]. The first step for both types is to develop an impact map (also called theory of change or logic model) with the stakeholders. The impact map shows the relationship between inputs, outputs and outcomes, and allows for the identification of indicators to measure outcomes [17, 22]. The next step is attributing values to the outcomes, referred to as the monetisation. Monetising the social outcomes of an intervention is one of the main challenges of the method as some may be difficult to monetise (e.g., subjective wellbeing or improved self-esteem) [16, 23]. To do so, financial proxies are used: they provide an estimate of financial value for outcomes or benefits that have no market value. To choose the financial proxies, the SROI guide presents methods used in environmental and health economics, such as contingent valuation, revealed preference and hedonic pricing. It is also recommended to use cost savings or an increased income when appropriate [17]. For instances, changes in health care costs resulting from an alteration in an individual’s personal health as well as changes in personal income due to an alteration of employment status could both be used as financial proxies. Proxy databases have also been developed in the last decade to assist SROI practitioners in the valuation process [24, 25]. They provide financial proxies to monetise outcomes, including those that are particularly hard to value, such as an increase in confidence or improved relationships. For example, the HACT value bank [24] uses results of large national surveys to isolate the effect of various factors, such as increased confidence or good overall health, on a person’s wellbeing to reveal the amount of money needed to increase someone’s wellbeing by the same amount [26]. Finally, to establish the actual impact of an investment it is necessary to consider deadweight. Establishing deadweight means determining what would have occurred anyway and is therefore not attributable to the intervention [17, 22].

As mentioned, PAS interventions are associated with a broad range of benefits including health, economic and social impacts. Therefore, the SROI framework seems to be relevant to support improved understanding, measurement and reporting the value created by these interventions and to inform policy-making. A previous systematic review of SROI studies in public health concluded that SROI is “very relevant and applicable” and that “it aids identification of the most impactful, cost-beneficial and culturally sensitive public health interventions” ([13], p., 12). However, to date, there is no review that focuses on the SROI framework in PAS. To fill this gap in knowledge, this review aims to systematically identify and review studies using the SROI framework within the field of PAS and assess their quality.

## Methods

A systematic search of the literature was conducted in accordance with the Preferred Reporting Items for Systematic reviews and Meta-Analyses (PRISMA) Statement [27].

### Inclusion criteria

We included peer-reviewed and grey literature studies using the SROI framework to evaluate any intervention, program or policy for which PAS is the main component. To be included, studies needed to be published in English and discuss the conduct of the study. Table 1 reports the inclusion and exclusion criteria following PICOS.

**Table 1:** PICOS criteria for inclusion and exclusion of studies

Parameter	Inclusion criteria	Exclusion criteria
Population	Any population addressed by the intervention, program or policy	
Intervention	Intervention, program or policy for which PAS is the main component	PAS is not the main component of the study
Comparator	Usual practice including no intervention	
Outcomes	Health and social impacts of the PAS intervention, program or policy	
Study design	SROI framework	SROI framework is used partially and the SROI ratio is not reported; Pilot and feasibility study; Abstract

### Search strategy

The search was comprised of two main steps: first, peer-reviewed articles were identified, and second, grey literature articles were identified. For both, search results were downloaded. Duplicates were removed and two reviewers independently screened the titles and abstracts of the articles following the criteria specified in Table 2. Articles that did not match the inclusion criteria were excluded. Finally, the full-text versions of the remaining articles were obtained and assessed for eligibility, using the same criteria.

For peer-reviewed articles, we searched PubMed, Scopus and Web of Science from January 1996 to September 2018. January 1996 was chosen because the first recorded SROI report was published during that year [13]. After performing an initial exploration and review of identified studies, we decided to use the following search terms: (‘social return on investment’ OR ‘SROI’) AND (‘sport’ OR ‘physical activity’).

**Fig. 1:**
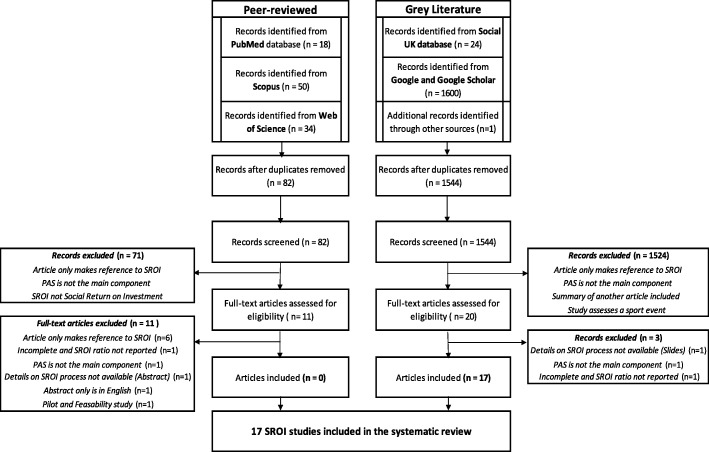
PRISMA flow diagram of study selection

To identify grey literature articles, we searched the Social Value International SROI-focused database, Google and Google Scholar from January 1996 to September 2018. For the SROI-focused database, ‘sport’ OR ‘physical activity’ were used as search terms. For the web search (Google and Google scholar), we employed the same search terms as for the peer-reviewed search. We also used the reference lists of identified articles to find other potential studies to be included. An email was sent to request the full report of one incomplete paper found online. The search strategy is summarised in Fig. 1.

### Data extraction and synthesis

Information was extracted from each article and classified in two summary tables: 1) Study and participant characteristics, and 2) Application of SROI and findings.

The first summary table, Study and participant characteristics, included: authors, year of publication, type of organisation commissioning the study, type of analyst performing the study, country where the study was conducted, type of PAS intervention, setting of the study and target population. We used four categories to classify the different types of PAS interventions: (1) Preventive – primary, (2) Preventive – secondary and tertiary [28], (3) Sport for development [29] and (4) High-performance sport.

The second summary table, Application of SROI and findings, included: SROI type (forecast or evaluative), purpose of the study, stakeholders included, study design, use of a proxy database (yes or no), type of outcomes and SROI ratio. Classifying outcomes was challenging given the lack of standardisation within the field. Therefore, to report the type of outcomes considered, we noted all indicators used in the studies and built categories (e.g., physical health, levels of social trust) in an iterative process. Afterward, we classified these outcome categories using the “indicator groups” presented in Krlev et al. [5]. Each ‘indicator group’ addresses a distinct type of outcome and an additional group covers negative outcomes. Each ‘indicator group’ with its respective abbreviation is listed as follows: Personal Resources: PR Community Resources: CR Regional Resources: RR Organisational Resources: OR Public Resources: PuR Societal Resources: SR Environmental Resources: ER Negative Outcomes: NO [15].

Stakeholders are defined as “people or organisations that experience change or affect the activity, whether positive or negative, as a result of the activity being analysed” ([17], p., 20). To classify stakeholders, we used the following classification ([13], p., 586):

i. Beneficiaries: users, those who experience the outcomes of an intervention.
ii. Implementers: includes project managers, suppliers and subcontractors.
iii. Promoters: those who provide support and a conducive environment for implementation of the intervention.
iv. Funders: those who finance the project.

### Quality assessment

To assess the quality of included articles the framework developed by Krlev et al. [15] was used. This framework consists of five dimensions (Transparency about why SROI was chosen; Documentation of the analysis; Study design and approximation of deadweight; Precision of the analysis and Reflection of the results) split into 12 criteria (Add. File 2). One point was awarded for each criterion that was considered ‘present’. If the criterion was ‘missing’ or ‘could not be ascertained’, no point was given. Papers were classified into high-quality (study scored ≥7) and low-quality (study scored < 7) [13, 15]. We used this quality assessment framework to perform comparisons between quality assessments completed in previous reviews of SROI studies.

## Results

### Study selection

We screened a total of 1626 titles. During abstract screening we excluded 1595 records. Key reasons for exclusion were: the paper refers to SROI without using it; PAS was not the main component of the assessed intervention, program or policy; SROI did not stand for Social Return on Investment; and the paper was a summary of another screened paper. We screened 31 full-text papers. The total number of SROI studies included in the present review is 17. All included studies were retrieved from grey literature search (Fig. 1). One included study [30] was peer-reviewed, yet identified through Google Scholar.

### Study and participant’ characteristics

The 17 included studies were published between 2010 and 2018. Thirteen were conducted in the UK [31–43], two in Australia [44, 45], one in Turkey [46] and one in South Africa [30]. Seven studies were performed by private consulting firms [31, 33, 35–37, 43, 46], four in an academic setting [39, 42–44], two by a sport organisation [41, 45] and one by a partnership between a university health board and a sport organisation [40]. Finally, three studies were performed by a social enterprise or a registered charity [24, 32, 38] (Table 2).

**Table 2:** Studies and participant characteristics

Ref.	Authors	Year of publica-tion	Type of organisation commissioning the study	Type of analysts performing the study	Country where the study was conducted	Setting of the PAS intervention	PAS intervention type	Target population of the PAS intervention
31	Baker Tilly	2010	Charity	Consulting firm	UK	Regional	Preventive - primary	General population
32	Lobley N & Carrick K	2011	Voluntary organisation	Social enterprise and independent charitable company	UK	Community	Preventive – sec. and tertiary	Individuals with long-term medical or mental health issues or elderly people
33	Baker Tilly	2013	Company limited by guarantee with charitable status	Consulting firm	UK	Regional	Preventive - primary	General population
34	Carrick K	2013	Public agency	Social enterprise and independent charitable company	UK	Municipal	Preventive - primary	Groups of city residents least likely to take regular exercise
35	ICF GHK	2013	Public agency	Consulting firm	UK	Regional	Preventive - primary	14- to 25-year-olds who are not particularly sporty
36	Charlton C	2014	Public agency	Consulting firm	UK	Regional	Preventive - primary	14- to 25-year-olds who are not particularly sporty
37	Butler W & Leathem K	2014	Charity	Consulting firm	UK	Community	Sport for development	Young people (under 17) from challenging neighbourhoods
44	Centre for Sport and Social Impact (CSSI), La Trobe University	2015	Public agency	Research/Academia	Australia	Community	Preventive - primary	Participants of typical community football club
46	Ozgun SC	2016	Private firm	Consulting firm	Turkey	School	Sport for development	Girls 10–12
38	New Economics Foundation and TCV	2016	Community volunteering charity	Consulting firm and Charity	UK	Community	Preventive – sec. and tertiary	Individuals coming from deprived background and with physical or mental health problems
39	Davies L, Taylor P, Ramchandani G, & Christy E	2016	Public agency	Research/Academia	UK	National	Preventive - primary	General population
40	Chin C	2016	Public agency	Partnership of a University Health Board and a Sport Organisation	UK	Regional	Preventive – sec. and tertiary	Disabled people
41	Hopkinson M	2016	Public agency and Charity (partnership)	Research consultant for a Sport Organisation (public)	UK	Community	Sport for development	Young people (14–25) living in disadvantaged communities across the UK
45	Darlington C.	2014	Not for profit Sport Association	Sport Organisation	Australia	Regional	High performance sport	High performance athletes
42	Baker C, Courtney P, Kubinakova K, Ellis L, Loughren E, & Crone D	2017	Public agency	Research/Academia	UK	Regional	Preventive – primary	General population
43	Regeneris Consulting	2017	Charity	Consulting firm (Pro Bono)	UK	Community	Sport for development	People with multiple and complex needs of all ages, from primary schoolchildren to prisoners
30	Sanders B & Raptis E	2017	Public agency	Research/Academia	South Africa	Community	Sport for development	Unemployed youth (18–30)

Amongst the studies included in the review, 15 were evaluative and two [30, 42] were forecast and evaluative. Only one study [40] used the SROI framework to inform decision-making. Three studies [35, 37, 41] reported its use for improved understanding of the impact and value created and five [30, 37, 38, 42, 46] used it to identify areas for improvement. Nine studies [30–34, 39, 43–45] used it exclusively in order to show and highlight the value created by the PAS intervention. One of these studies [32] mentioned securing funding as an objective of the SROI analysis. Most studies [30–36, 38, 40, 41, 45, 46] included all stakeholders in the analysis. To estimate deadweight, three studies [37, 42, 43] employed a before-and-after design, one study [44] used a comparison group and 12 [30–36, 38, 40, 41, 45, 46] used opinions and assumptions of stakeholders. Finally, one study [39] relied on a data analysis based on a systematic review of literature and consultation of academic experts. To monetise outcomes, a little less than half (47%) of the studies [30, 35, 36, 39–41, 43] used financial proxy databases (Table 3).

PAS interventions related mostly to Primary prevention [31, 33–36, 39, 42, 44], followed by Sport for development [30, 37, 41, 43, 46], Secondary and Tertiary prevention [32, 38, 40], and High-performance sport [27]. Primary prevention interventions targeted general population [31, 33, 39, 42, 44] or non-active groups [34–36] in community [44], regional [31, 33, 35, 36, 42], municipal [34] or national settings [39]. Sport for development interventions targeted people with multiple and complex needs of all ages [43], girls [46], disadvantaged youth [37, 41] and unemployed youth [30] in communities [30, 37, 41, 43] or school settings [46]. Secondary and tertiary prevention interventions targeted individuals from disadvantaged backgrounds and with physical or mental health problems [38], disabled people [40] or individuals with long-term medical or mental health issues [32] within communities [32, 40] or regional settings [40]. Finally, the high-performance sport intervention [E] targeted athletes in a regional setting.

### Quality assessment

Out of a maximum of 12 points on the Krelv et al. quality assessment framework, scores ranged from 2 to 11 with a median of 7 and mean of 6.5. About half (53%) of the studies got a score of 7 or higher [32, 34, 35, 37, 40–43, 46], which is defined as a good score [13, 15]. Of the five framework dimensions, Study design was the least successful. No study used a control group, and only 18% of the studies conducted a comparison of observations before and after intervention. Another weakness of the included studies was transparency regarding the choice of SROI. Indeed, the majority of studies explained SROI but failed to discuss the reasons for adopting this method over another. Also, only half of the studies mentioned using an impact map and less than half (47%) used quantitative methods to measure outcomes. As for the strong dimensions, Reflection of the results was the most successful. Between 65 and 76% of the studies discussed limitations of their analyses, interpreted the SROI ratio obtained and performed at least one sensitivity analysis. Finally, 76% of the studies had valid and comprehensive proxies.

### Application of SROI and findings

One of our key finding was the type of outcomes measured and monetised in the studies. Due to a lack of standardisation within the field we decided to use the groups presented in Krlev et al. [15] to classify the various outcomes. Each group addresses a distinct type of outcome and consists of several categories. For example, Personal Resources (PR) outcomes were found in all studies under different categories, including the following: Physical health, Mental health and wellbeing, New knowledge and skills, Self-efficacy, Educational attainment and Self-esteem. Community Resources (CR) outcomes were found in 65% of the studies under different categories, including: Social contacts and improved relationships, Social trust, Reduction of anti-social behaviour and Community role models. Regional Resources (RR) outcomes were found in 35% of the studies under the following categories: Economic development and Family retention in regions. Organisational Resources (OR) outcomes were found in 29% (categories: Increased capacity, Productivity gain), Public Resources (PuR) in 24% (categories: Reduced obesity, Fiscal benefits) and Societal Resources (SR) in 12% of the studies (categories: Better understanding of ethnicity and disability, Interaction with others from different cultural and social backgrounds and Awareness of gender inequalities). None of the studies measured Environmental resources or Negative outcomes. The High-performance sport PAS type is associated with the lowest range of outcomes (PR, CR, RR), followed by Secondary and tertiary prevention (PR, OR, PuR, CR). Primary prevention and Sport for development are both associated with the highest range of outcomes (PR, OR, PuR, RR, CR, SR).

Not all of the outcomes measured in the studies were monetised. For example, Physical health was measured in 14 studies but only monetised in 9. Outcomes were not calculated in the SROI ratio if they were not also monetised, yet they were still presented as relevant information in the studies. Reasons for not monetising outcomes included the difficulty with identifying a relevant financial proxy and insufficient evidence for an outcome to be properly valued [16]. The most monetised outcome group was Personal Resources (in 82% of the studies), followed by Community Resources (in 41% of the studies) and Regional Resources (in 35% of the studies). In terms of outcome categories, Physical Health, included in the PR outcome group, was the most measured (82% of the studies used indicators to measure it) but Mental Health and wellbeing was the most monetised (monetised in 65% of the studies) (Add. file 3).

We also found a pronounced difference in SROI ratio between studies: it varied from 1.7:1 to 124:1. A ratio of 1.7:1 indicates that an investment of $1 delivers $1.7 of social value. The lowest reported ratios (1.7:1) were for a sport-based youth development program and a high-performance program. The highest (124:1) was for a PAS program for disabled people. We calculated SROI ratio average for each PAS type. The highest (44:1) is associated to Secondary and tertiary prevention, followed by Sport for development (5.9:1), Primary prevention (5.6:1) and High-performance (1.7:1).

**Table 3:** SROI application and findings

Ref.	SROI type	Purpose of the SROI study	Stakeholders included in the analysis	Study design	Use of a proxy database	Outcome groups included in the study	SROI
31	Evaluative	Evaluate the benefits generated by the services	Beneficiaries, implementers, funders, promoters	Post-intervention data collection + opinions and assumptions of the stakeholders	No	PR, OR, PuR	Not mentioned
32	Evaluative	Evaluate the social value created by the project, prove the wide impacts of the project and hope to secure continued funding	Beneficiaries, implementers, funders, promoters	Post-intervention data collection + opinions and assumptions of the stakeholders	No	PR, OR	4:1
33	Evaluative	Evaluate the benefits generated by the services	Beneficiaries, implementers, funders, promoters	Post-intervention data collection + opinions and assumptions of the stakeholders	No	PR, RR	Not mentioned
34	Evaluative	Identify and value the benefits delivered by the intervention	Beneficiaries, implementers, funders, promoters	Post-intervention data collection + opinions and assumptions of the stakeholders	No	PR, CR, SR, OR, PuR	8:1
35	Evaluative	Understand the impact of the program and identify key lessons to improve processes, approaches and future impact.	Beneficiaries, implementers, funders, promoters	Post-intervention data collection + opinions and assumptions of the stakeholders	Yes	PR, CR, OR	7.5:1
36	Evaluative	Missing	Beneficiaries, implementers, funders, promoters	Post-intervention data collection + opinions and assumptions of the stakeholders	Yes	PR, PuR, CR, RR, OR	3:1
37	Evaluative	Recognise and understand the social value created, identify improvements and potential areas of negative social value, inform stakeholders and account to funders.	Beneficiaries, implementers, funders, promoters	Before-and-after	No	PR, CR	4.4:1
44	Evaluative	Determine the social value of a typical community football club, specifically its social, health and community impact.	Beneficiaries, funders	Comparison group	Not mentioned	PR, CR, RR	4.4:1
46	Evaluative	Determine the impacts of the project, identify the most productive and inefficient parts, use the resources in the most efficient way, promote and extend the project.	Beneficiaries, implementers, funders, promoters	Post-intervention data collection + opinions and assumptions of the stakeholders	No	SR, PR	12.5:1
38	Evaluative	Demonstrate the impact on health, wellbeing and employability. Create systematic monitoring and measurement of outcome. Identify opportunities for learning, change and improvement.	Beneficiaries, implementers, funders, promoters	Multiple data collection during + opinions and assumptions of the stakeholders	Not mentioned	PR, CR	4:1
39	Evaluative	Enable policy makers to present a case for supporting investment in sport by demonstrating its wider contribution and value to society.	Beneficiaries, implementers, funders, promoters	Data analysis based on a systematic review of literature and consultation of academic experts in the field of health, crime, education and social capital	Yes	PR, CR	1.9:1
40	Evaluative	Examine whether the intervention is a cost effective one that should be rolled out across the country.	Beneficiaries, implementers, funders, promoters	Post-intervention data collection + opinions and assumptions of the stakeholders	Yes	PR, CR, PuR	124:1
41	Evaluative	Understand and evidence the broad value of developing high-quality coaches.	Beneficiaries	Post-intervention data collection + opinions and assumptions of the stakeholders	Yes	PR, CR, RR	3:1
45	Evaluative	Provide a result of the social return on investment (SROI) that MWAS created for stakeholders.	Beneficiaries, implementers, promoters, funders.	Opinions and assumptions of the stakeholders	No	PR, CR, RR	1.7:1
42	Forecast and Evaluative	Understand and value the changes that occurred because of projects implemented with AT funding, and develop delivery and evaluation blueprint as a resource for the other organisations and similar programs.	Beneficiaries, implementers, funders, promoters	Before-and-after	Yes	PR, CR	7.3:1
43	Evaluative	Provide an economic and social impact assessment of the organisation.	Beneficiaries	Before-and-after	Yes	PR	9:1
30	Forecast and Evaluative	Evaluate a sport for development and peace intervention.	Beneficiaries, implementers	During-and-after + opinions and assumptions of the stakeholders	Yes	PR, RR	1.7:1

## Discussion

This systematic review identified a total of 17 SROI studies within the PAS field. Studies were published between 2010 and 2018, suggesting that the use of SROI in this field is relatively recent. Nearly all of the studies (94%) come from the grey literature and were largely conducted in the UK (76%) by private consulting firms (41%). Secondary and tertiary prevention PAS intervention demonstrates, on average, the highest SROI ratio but Primary prevention and Sport and development interventions are associated with the largest scope of outcomes. About half of the studies (53%) were identified as high-quality.

Regarding outcomes, our results highlight the broad range of PAS impacts to society as well as efforts deployed in the field to measure and valorise them. Although the Personal Resources outcomes are the most measured and monetised, Community Resources outcomes (such as Social contacts and Social trust) are emphasised much more in PAS studies than in general SROI studies [15]. Furthermore, PAS interventions were associated with a large range of outcome categories, from physical health to economic development, revealing that PAS can contribute value to society across multiple individual and populational outcomes. It highlights the relevance of the method to improve the understanding, measurement and reporting of the contributions of PAS to society in a way that traditional economic evaluation framework cannot provide [13, 20]. To further understand how the value is distributed across outcomes, future studies could make it more explicit how each outcome category contributes to the total social value created. This could be done by reporting the percentage of the total value attributed to each outcome category. Conversely, a weakness of the field is that none of the studies included negative outcomes. This weakness has also been noted in previous SROI reviews [15, 23].

Reported PAS SROI ratios vary between 1.7:1 and 124:1. These ratios suggest that every analysed PAS intervention provides a positive return on investment to society. If we exclude the highest ratio and select only the high-quality studies, ratios vary between 3:1 and 12.5:1. In comparison, a SROI review in public health reported ratios varying between 1.1:1 and 65:1 [13]. A possible explanation for the highest range in public health might be the number of studies included in their review: 40, compared to 17 in this current review. The concise format of the value created for invested money in the form of a ratio is a strength of the SROI method: it is an effective communication tool and can provide external accountability [47]. However, because of a large heterogeneity in the application of the SROI method, it is not possible to use solely the ratios to compare and identify the intervention generating the greater social value [13, 16–19, 48].

The preponderance of UK SROI studies has been noted in previous SROI reviews [13, 15]. A possible explanation is the credence given to the method by the UK government and policymaking bodies [15, 18, 23] as well as the introduction of the UK Social Value Act in 2013 [49]. The Social Value Act increased pressure for public services, including Sport and Recreation, to procure services that have social, economic and environmental value [18]. Mainly private consulting firms conducted the SROI studies (41%), which was followed by academics (30%). This is possibly due to the limited expertise of Sport and recreation service managers to undertake a SROI analysis [18]. A recent review [23] noted that, to date, academics have been slow to adopt the SROI framework in the evaluation of health and social care interventions. However, in this current review, 30% of the studies were performed by academics (compared to 7% in all SROI studies [15]) suggesting that academics in the field might be early adopters of the method. This difference does not appear to have made an impact on the quality and provenance (peer-reviewed or grey literature) of studies: the studies performed by academics did not received a high-quality score on average (6.2 out of a maximum of 12), and 94% of the studies were classified as grey literature.

Overall, SROI studies in the PAS field appear to be of lower quality than those conducted in the field of public health but slightly superior to SROI studies in general: 53% of the SROI studies obtained a high-quality score in PAS vs. 70% in public health [13] and 46% for SROI in general [15]. The quality assessment highlighted some weaknesses of the field, including no mention of a clear rationale for the choice of SROI framework and a lack impact map (or logic model) usage. This latter weakness is surprising as the impact map is a critical step of the method. The quality assessment also exposed some strengths: 71% of the studies performed sensitivity analysis (compared to 47% in all SROI studies [5]) which increases confidence in the SROI ratios presented [23]. Furthermore, 76% used valid and comprehensive financial proxies (58% in all SROI studies [15]). As for financial proxies, almost half of the studies (47%) used financial proxy databases (HACT or GVE) to select them. Using these databases improve standardisation in the monetisation process, which can increase the robustness of a study by removing subjectivity related to valuation methods. Yet, it prevents the tailoring of the SROI analyses, thus potentially affecting its validity [47]. For example, one of the included studies analysed a South African PAS intervention and used databases to identify financial proxies that were calculated from UK data. Indeed, financial proxies from a different country may not reflect the value of outcomes for the actual beneficiaries. One approach for future SROI studies to improve the robustness of financial valuations may be to use the Delphi method, which triangulate valuations of beneficiaries with a panel of “experts” [50].

Regarding the purpose of studies, most (53%) used the SROI framework exclusively to highlight the social value created by interventions, without intention to inform a decision, even if the framework has been created essentially as a tool to inform practical decision-making [14, 17, 48]. In the context of the Social Value Act, reinforcing a provider’s position in a competitive environment [51] and attracting funding [52] might be key motivations for conducting a SROI, as well as enabling commissioners to make well informed decisions in the procurement process [18]. This is perhaps an explanation of the current state of the field, which appears to be driven more by demonstrating the value created by an intervention rather than using the framework as a practical decision-making tool.

A SROI analysis is a multifaceted evaluation tool, involving mapping, reporting and monetising outcomes to establish the impact of an intervention in relation to its costs. Making robust financial valuation when monetising outcomes is often cited as one of the main challenges of the method [16, 50, 53]. However, robust financial valuation can only partly improve the robustness of the SROI if outcomes are not properly measured and if deadweight is not robustly established using a proper study design. The latter appears to be the main limitation of SROI studies in the PAS field. Indeed, none of the included studies used a control group, only one used a comparison group, less than 20% used a before-and-after design and most studies used opinions and assumptions of stakeholders to estimate deadweight. To accurately determine the impact of interventions through SROI studies, Banke-Thomas et al. [13] emphasised the importance of using objective study designs such as case-control, before-and-after comparisons or a stepped-wedged cluster randomised trial [50]. Given organisational, financial and ethical constraints, non-academic organisations face significant limitations in adopting such study designs. However, studies conducted in an academic setting (30% of the studies in this review) are well positioned to use designs such as the stepped-wedged cluster randomised trial [50], to adopt methods that lend themselves to natural experiments or to conduct non-experimental observational studies [54]. To operationalize these study design in the SROI method, it has been suggested that a SROI analysis should start with a forecast-type SROI to model and predict the potential social impact of an intervention prospectively. This forecast-type study would allow researchers to identify data needs as well as to plan the evaluation process and study design to account for deadweight. An evaluative-type SROI would be performed after the implementation to retrospectively account for actual outcomes that have occurred [17, 50].

### Strengths and limitations of the current review

The strengths of the current systematic review include the replicable search strategy within both peer-reviewed and grey literature databases, the in-depth synthesis focusing on study and participant characteristics and the quality assessment that allowed for comparisons to previous reviews. Our review also has limitations. First, non-English language articles were not included. Second, some criteria of the Krlev et al. assessment quality framework, such as Transparency about why SROI was chosen, were difficult to judge because of their subjectivity. We followed the criteria to the best of our ability according to their description [15] and applied them consistently to all studies included in this review. However, it is possible that we were more or less strict with certain criteria as compared to Krlev et al. [15] or Banke-Thomas et al. [13], which may affect the reliability of our comparisons.

## Conclusion

To our knowledge, this is the first review to systematically identify studies using the SROI framework within the field of PAS. This study highlighted its relevance to further understand, evidence and value the wide benefits of PAS to society. In this regard, the SROI framework can be a useful tool to inform policy-making related to PAS investment. However, further research is needed to improve the robustness of the SROI application in the field. Study quality in the field would benefit from using an impact map (or logic model) and reporting negative outcomes. Academics could also play an important role in improving the quality of SROI studies by integrating the study design to account for deadweight. The application of the SROI method in the PAS field is relatively recent, and thus further research would be beneficial to promote its potential for policy-making bodies in the field.

## Supplementary information

**Additional file 1.** PRISMA checklist

**Additional file 2.** Quality assessment

**Additional file 3.** Outcome groups and categories

## Data Availability

The dataset supporting the conclusions of this article are included within the article (and its Additional files).

## Abbreviations

CBA: Cost-benefits analysis
CEA: Cost-effectiveness analysis
CR: Community resources
CUA: Cost-utility analysis
ER: Environmental resources
NCD: Non-communicable diseases
NO: Negative outcomes
OR: Organisational resources
PAS: Physical Activity and Sport
PR: Personal resources
PuR: Public resources
RR: Regional resources
SR: Societal resources
SROI: Social return on investment
